# Nutritional Profile, Lifestyle Characteristics, and Intestinal Inflammation in Lebanese Adults Suffering from Food Hypersensitivities: A Case–Control Study

**DOI:** 10.3390/nu18142264

**Published:** 2026-07-10

**Authors:** Gregory Hage, Yonna Sacre, Marcel Hajj, Nicole Fakhoury-Sayegh

**Affiliations:** 1 Doctoral College, Holy Spirit University of Kaslik, Jounieh P.O. Box 446, Lebanon; 2 Department of Nutrition and Food Science, Faculty of Arts and Sciences, Holy Spirit University of Kaslik, Jounieh P.O. Box 446, Lebanon; 3 Hajj Medical Center-Medical & Dental Clinics, Green Zone A Building 71 Ground Floor, Naccache P.O. Box 1201, Lebanon

**Keywords:** food hypersensitivity, food allergy, food intolerance, IBS

## Abstract

**Background:** Food hypersensitivity is frequently associated with gastrointestinal and systemic manifestations. This study aimed to evaluate the clinical, nutritional, biochemical, lifestyle characteristics, and stress levels of Lebanese adults with food hypersensitivity (cases) (*n* = 378) compared with controls (*n* = 397) (absence of food hypersensitivity). **Methods:** A case–control study was conducted among 775 Lebanese adults, including participants with self-reported food allergies and/or food intolerance and controls. Sociodemographic, clinical, and lifestyle data were collected. Dietary intake was assessed using validated dietary assessment tools. Biochemical parameters (*n* = 775), stool analyses (*n* = 297), and fecal calprotectin were evaluated. **Results:** Overall, 378 participants (48.8%) were classified as having food hypersensitivity. Dermatological, nasal, respiratory, and gastrointestinal symptoms were significantly more frequent among cases than controls (*p* < 0.05). Autoimmune diseases were more prevalent among cases. Daily energy and nutrient intake differed significantly between groups, with cases generally reporting lower intakes than controls. Cases exhibited substantially lower serum vitamin D, vitamin B12, and hematocrit levels. In binary logistic regression models, significant correlations were observed alongside key antioxidant and immunomodulatory micronutrients: vitamin C (OR = 0.604; 95% CI: 0.390–0.937), dietary vitamin D (OR = 0.079; 95% CI: 0.011–0.565), vitamin E (OR = 0.085; 95% CI: 0.013–0.568), and iron (OR = 0.025; 95% CI: 0.002–0.291). **Conclusions:** Biochemical and nutritional differences were observed despite generally adequate dietary intake. Reduced intakes of essential micronutrients, including antioxidant vitamins (vitamins C and E), vitamin D, and the essential mineral iron, emerged as primary independent predictors of food hypersensitivity. These findings highlight the core role of localized intestinal inflammation, specific dietary avoidance behaviors, and systemic immune modulation.

## 1. Introduction

Food hypersensitivities, encompassing both immunologically mediated food allergies and non-immunological food intolerances, represent an important and growing public health concern worldwide [1]. The prevalence of food allergies is estimated to range between 2 and 10% of the population. Food intolerances are thought to affect an even greater proportion, although precise figures are often underreported due to diagnostic challenges [2]. These conditions may lead to a wide spectrum of clinical manifestations, from mild gastrointestinal discomfort to life-threatening anaphylaxis [3]. Beyond the immediate medical implications, food hypersensitivities have profound impacts on patients’ health, daily functioning, and dietary behaviors [3].

Strict avoidance of offending foods is effective in preventing adverse reactions but can alter dietary patterns and predispose individuals to nutritional inadequacies, including deficiencies in calcium, vitamin D, vitamin B12, iron, zinc, and high-quality protein [3,4]. Evidence from Europe, North America, and parts of Asia supports these concerns, showing lower intakes of key micronutrients, deviations from recommended dietary patterns, and greater reliance on restricted or selective diets among affected individuals compared with healthy controls [5,6,7]. Common allergenic foods such as cow’s milk, eggs, wheat, soy, nuts, and seafood are major contributors of essential macronutrients and micronutrients, and exclusion of these foods may further compromise nutritional status [4,8]. In children, these deficiencies can impair growth and development, while in adults they may exacerbate chronic disease risks and reduce overall nutritional health [3,5]. Furthermore, individuals often compensate for food avoidance by increasing intake of processed alternatives, which may be higher in sugars, fats, or additives, thus altering overall diet quality [8].

Beyond generic global labeling and supply chain impediments, the Lebanese context presents a highly specific constellation of compounded structural and socio-cultural determinants that uniquely shapes adult nutritional and clinical vulnerability. Centrally, the lack of a centralized national surveillance system for food safety leaves consumers highly exposed to unmonitored microbial and environmental shifts, while a deeply entrenched culture of medical self-prescribing drives widespread, unmanaged dietary elimination strategies. Furthermore, distinct Levantine lifestyle patterns—such as dense urban architectural barriers that minimize natural solar exposure despite abundant seasonal sunshine, coupled with a high regional prevalence of poly-tobacco use including traditional argileh smoking, and dietary habits characterized by increased consumption of wheat-based products, dairy foods, legumes (particularly beans), alcohol, frequent social gatherings, and large meal portions —may directly compound mucosal barrier fragility and alter systemic micronutrient availability [9,10]. Consequently, a dedicated Levantine study is vital, as these localized environmental and behavioral dynamics generate hidden nutritional deficiencies and low-grade intestinal inflammation that cannot be accurately extrapolated from Western or East Asian epidemiological models.

Accordingly, this study aimed to examine the nutritional profiles, stress levels, and lifestyle characteristics of the cases compared with controls. We hypothesized that individuals with food hypersensitivities could exhibit lower nutrient intakes compared to controls, leading to macronutrient and micronutrient inadequacies and clinical problems. The ultimate goal of the study was to provide evidence to support clinical dietary counseling and inform public health strategies in Lebanon.

## 2. Materials and Methods

### 2.1. Study Design and Population

This case–control study included 775 Lebanese adults, comprising 378 individuals with food hypersensitivity (cases) and 397 controls without known food hypersensitivity. Participants were recruited consecutively from July 2024 to June 2025 among patients attending an outpatient clinic at a Medical Center in the Beirut region for routine health check-ups. All eligible patients seen during the study period were invited to participate, and written informed consent was obtained at enrollment. Participants were drawn from all Lebanese governorates to ensure broad geographic representation.

### 2.2. Eligible Population

The study sample included consecutive visits of patients coming from all Lebanese governorates to this outpatient clinic. A convenience method of recruitment was used. To minimize selection bias, participant recruitment followed a strict face-to-face protocol. The main clinical researcher directly approached potential participants in the waiting area of the outpatient clinic. Eligible individuals were those presenting exclusively for standard, routine annual physical check-ups. To eliminate advertisement-driven or self-selection biases, no external media, digital campaigns, public notices, or physical flyers were utilized at any stage of recruitment. This direct, clinic-based convenience sampling strategy ensured that both cohorts were drawn from a population presenting for routine health maintenance rather than individuals actively seeking tertiary care for acute gastrointestinal or allergic distress. The inclusion criteria differed distinctly between the two study arms and were defined as follows:

- Cases: Eligible cases included Lebanese men and women aged between 18 and 63 years old who possessed a verified, formal, historical physician-diagnosed food allergy and/or food intolerance.
- Controls: Eligible controls included Lebanese men and women within the same age bracket (18–63 years) defined by the complete lifetime absence of any known, historical, or suspected food hypersensitivities.

Additional shared inclusion criteria for both groups included non-pregnancy among women and the absolute completeness of all survey, dietary, and clinical data except for stool analysis where we had a completeness rate of 38.3% of all participants. Shared exclusion criteria for both groups included active gastrointestinal malignancies, known eating disorders (e.g., anorexia or bulimia nervosa), or the active use of potent immunomodulating drugs, prebiotics, probiotics, vitamins or mineral supplements, or nutraceuticals or long-term medications that could systematically alter gastrointestinal function or baseline nutritional status.

To ensure methodological rigor, a strict chronological blinding and sequencing workflow was enforced during enrollment. All participants were consecutively approached and enrolled at baseline upon presenting for their routine health check-ups, prior to undergoing or receiving results from their scheduled annual laboratory and allergy screening batteries. The comprehensive 157-item Food Frequency Questionnaire (FFQ) and psychometric interviews were completed entirely during this initial baseline phase while participants remained unclassified. Final group stratification into cases (physician-verified hypersensitivity) or controls was performed retrospectively only after all diagnostic markers—including specific IgE, skin prick tests, microarray parameters, and hydrogen breath tests—were finalized and reviewed in the medical charts.

### 2.3. Ethical Considerations

**Figure 1:**
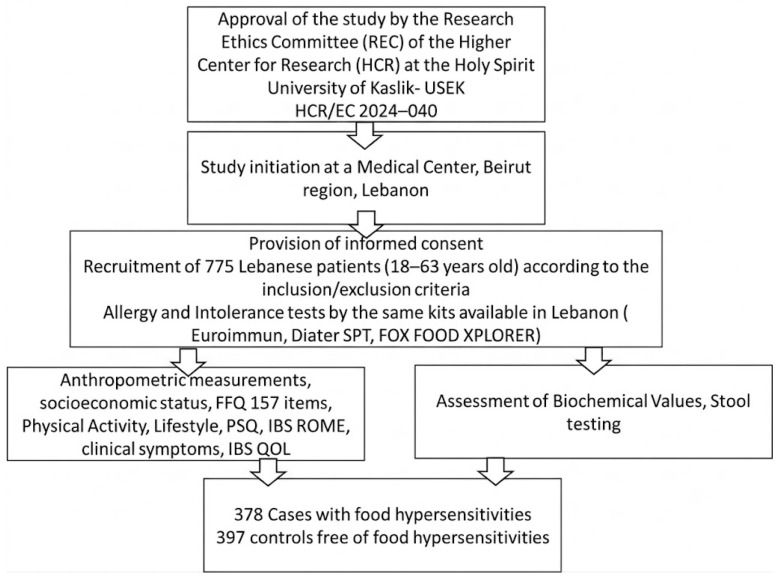
Flowchart for selection and enrollment of study subjects (cases and controls).

The study protocol was approved by the Research Ethics Committee (REC) of the Higher Center for Research (HCR) at the Holy Spirit University of Kaslik-USEK (HCR/EC 2024-040). All participants provided written informed consent before enrollment. Confidentiality of personal and medical information was strictly maintained throughout the study (Figure 1).

### 2.4. Sociodemographic and Lifestyle Assessment

Participants completed a structured questionnaire to gather sociodemographic information, including age, sex, governorate of residence, educational attainment, employment status, and marital status. Lifestyle characteristics were also recorded, notably smoking behavior and type of tobacco use (cigarettes, argileh, or e-cigarettes per day. Physical activity levels were also studied using the IPAC questionnaire [11]. Perceived stress was evaluated using the Arabic version of the Perceived Stress Questionnaire (PSQ-30), a 30-item instrument originally developed by Levenstein et al. (1993) [12,13] that effectively differentiates stress dimensions into specific clinical subscales. The Arabic version was selected to ensure optimal comprehension and cultural appropriateness for the study population and to minimize potential language-related misunderstandings. The psychometric robustness and internal consistency of the PSQ-30 within our study sample were verified through reliability analysis, demonstrating strong internal consistency with a calculated overall Cronbach’s alpha (α) of 0.84 [13].

### 2.5. Clinical Assessment

Medical history included hypertension, type 2 diabetes, dyslipidemia, cardiovascular, renal, hepatic, and pulmonary diseases, cancer, immunodeficiency, and autoimmune disorders. Participants were further stratified according to the presence of gastrointestinal symptoms, allergies, and food intolerances. Physical examinations were conducted to obtain anthropometric measurements, including height (m), weight (kg), and body mass index (BMI) (kg/m^2^). All physical measurements were performed by the same investigator and measured three times consecutively. The average of the three measurements was recorded. Height and weight were measured with participants standing barefoot and wearing light clothing, using a wall-mounted stadiometer and a mechanical scale, respectively. Weight was recorded to the nearest 0.5 kg and height to the nearest 0.5 cm.

### 2.6. Food Hypersensitivity Assessment

Laboratory assessment of food hypersensitivity included the following tests. The measurement of total IgE and food-specific IgE used EUROIMMUN kits (Medizinische Labordiagnostika AG, Lübeck, Germany, 2024). The skin prick testing used Diater reagents (Diater Laboratorio de Diagnóstico y Aplicaciones Terapéuticas S.A., Madrid, Spain, 2024). The IgG-based food intolerance testing used the FOX Food Xplorer microarray (Macro Array Diagnostics [MADx], Vienna, Austria, 2024). The screening for celiac disease was performed by measuring IgA anti-tissue transglutaminase (tTG-IgA) and/or anti-endomysium antibodies using CE-marked ELISA and indirect immunofluorescence kits (Immundiagnostik AG, Bensheim, Germany, and EUROIMMUN Medizinische Labordiagnostika AG, Lübeck, Germany, 2024). Lactose intolerance was assessed using a hydrogen breath test (HBT) system (Sleuth Hydrogen Breath Test System, Breathe E-Z Systems, Inc., Leawood, KS, USA, 2024).

### 2.7. Biochemical and Stool Analyses

Biochemical analyses were performed using automated laboratory analyzers and commercially available diagnostic kits distributed in Lebanon. Hematological parameters including hemoglobin and hematocrit were measured using an automated hematology analyzer (Sysmex XN-1000 automated hematology analyzer (Sysmex Corporation, Kobe, Japan; software version 22).). Serum ferritin levels were determined using a Ferritin ELISA kit (DRG Instruments GmbH, Marburg, Germany, 2024). Serum vitamin B12 concentrations were measured using a Vitamin B12 ELISA kit (EUROIMMUN Medizinische Labordiagnostika AG, Lübeck, Germany, 2024). Serum folic acid concentrations were determined using a Folate ELISA kit (DRG Instruments GmbH, Marburg, Germany, 2024). Serum 25-hydroxyvitamin D levels were quantified using a 25-OH Vitamin D ELISA kit (EUROIMMUN Medizinische Labordiagnostika AG, Lübeck, Germany, 2024).

Stool analyses included coproculture (stool culture) for bacterial, fungal, and parasitic infections using standard microbiological culture methods. Fecal calprotectin levels were measured using a Calprotectin ELISA kit (Immundiagnostik AG, Bensheim, Germany, 2024). Stool analyses, including microscopic examination and fecal calprotectin quantification, were completed for a sub-cohort representing 38.3% (*n* = 297) of the total study population. The remaining 61.7% (*n* = 478) of participants did not provide stool specimens primarily due to logistical constraints, compliance factors, or an inability to return samples within the required clinical window.

### 2.8. Gastrointestinal Assessment and IBS Diagnosis

IBS diagnosis was determined using the Rome III criteria via a structured questionnaire. The Rome III framework was deliberately selected over the newer Rome IV criteria because it remains widely translated, culturally adapted, and extensively validation-tested in Arabic clinical settings across the Middle East. Furthermore, within epidemiological field and cross-sectional settings, the highly restrictive frequency thresholds mandated by Rome IV can overly suppress case-capturing rates, potentially underrepresenting individuals experiencing clinically significant, chronic functional symptoms [14].

### 2.9. Dietary Assessment and Nutritional Status

Dietary intake was assessed using a validated Food Frequency Questionnaire (FFQ) to estimate macronutrient and micronutrient intake [15]. FFQ data were analyzed using NutriLog software (version 3.20; Nutrilog SAS, La Calale, 2 rue du Grand Both, 17230 Marans, France) to calculate total energy intake/day, macronutrients/day; protein (g), carbohydrates (g), and fat (g), and micronutrients (vitamins and minerals)/day. Supplement consumption was additionally considered when estimating the daily intake of the selected items. To minimize reporting bias, dietary interviews were conducted before the diagnosis of food hypersensitivity. Biochemical nutritional status was evaluated through serum measurements of hematocrit (%), hemoglobin (g/dL), ferritin (ng/mL), folic acid (ng/mL), vitamin B12 (pg/mL), and vitamin D (ng/mL) (specific tests for all these parameters). To entirely prevent post-diagnostic behavior modification bias, the validated 157-item FFQ interviews were completed at baseline while participants were still fully blinded to their upcoming updated annual diagnostic and laboratory test results. Formally unblinded clinical group assignments (Cases vs. Controls) were only assigned after final medical chart reviews.

### 2.10. Microbiological and Inflammatory Assessments

Stool samples were collected for analysis to detect bacterial, fungal, and parasitic infections (stool culture, parasite, and calprotectin). Intestinal inflammation was assessed using fecal calprotectin testing, with positive results indicating underlying inflammation or dysbiosis [16].

### 2.11. Statistical Analysis

Descriptive statistics were computed using frequencies and percentages for categorical variables and means ± standard deviations (SD) for continuous variables. Geometric means (log_10_-transformed quantitative variables) were used when the data did not follow a normal distribution. Case–control comparisons were conducted using Chi-square tests for categorical variables and independent-samples *t*-tests for continuous variables. Binary regression analyses were additionally performed to evaluate associations between food hypersensitivity and selected clinical, biochemical, and nutritional variables. A *p*-value < 0.05 was considered statistically significant. All statistical analyses were performed using IBM SPSS Statistics for Windows, Version 23.0 (IBM Corp., Armonk, NY, USA).

To evaluate potential missing data bias, a sensitivity analysis was conducted comparing the baseline characteristics of participants with available stool data against those without, utilizing Chi-square tests for categorical variables and independent-samples *t*-tests for continuous variables.

## 3. Results

### 3.1. Study Population and Classification of Food Hypersensitivity

A total of 775 participants were included in the study. Among them, 378 individuals (48.8%) were classified as cases, while 397 participants (51.2%) served as controls. Within the case group, 178 participants (47.1%) reported food allergy, 82 (21.7%) reported food intolerance, and 13 (3.4%) reported both food allergy and intolerance. In addition, 105 participants presented with irritable bowel syndrome (IBS) either alone or in combination with food allergy or intolerance, whereas only four participants reported isolated IBS without associated food hypersensitivity. The distribution of participants according to food hypersensitivity classification is presented in Table 1.

Sociodemographic characteristics of cases and controls were summarized in Table 2. The distribution of sex was comparable between groups, with no statistically significant differences observed. Mean age (years) did not differ significantly between cases and controls. Participants were recruited from all Lebanese governorates, with Mount Lebanon and Beirut representing the largest proportions in both groups. Educational level, marital status, employment status, and household income were similar between cases and controls.

### 3.2. Anthropometric, Clinical, and Lifestyle Characteristics

Anthropometric measurements, including body mass index (BMI), did not differ significantly between cases and controls. The prevalence of chronic diseases such as hypertension, type 2 diabetes, dyslipidemia, cardiovascular disease, renal disease, liver disease, pulmonary disease, cancer, and immunodeficiency was comparable between groups. In contrast, autoimmune diseases were significantly higher among cases compared with controls.

The prevalence of smoking was significantly higher among cases than controls. No significant differences were observed between groups regarding physical activity. Detailed anthropometric, clinical, and lifestyle characteristics were presented in Table 3.

### 3.3. Biochemical and Microbiological Findings

Biochemical analyses showed significantly lower levels of hematocrit, hemoglobin, vitamin B12, and vitamin D among cases compared with controls (*p* < 0.05). Sex-stratified analyses indicated that the difference in hematocrit was significant among women but not men, while hemoglobin levels were significantly lower among men but not women. No statistically significant differences were observed for folic acid or ferritin levels between the two groups.

Microbiological stool examinations revealed significant differences between groups. Positive bacterial infections were more frequently detected among controls (17.2%) compared with cases (4.23%) (*p* = 0.001). In contrast, fungal infections (17.1% vs. 13.9%, *p* = 0.001) and parasitic infections (17.5% vs. 8.1%, *p* = 0.016) were significantly more prevalent among cases. Additionally, positive fecal calprotectin results were significantly more common in cases than controls (23.0% vs. 8.1%, *p* < 0.001).

A sensitivity analysis was performed comparing baseline characteristics between participants who provided stool samples (*n* = 297) and those who did not (*n* = 478). No statistically significant differences were observed between the two sub-cohorts across major demographic variables—including age (*p* > 0.05, independent-samples *t*-test) and sex, educational level, or governorate of residence (*p* > 0.05, Chi-square tests). This confirms that the missing stool data occurred at random and introduces negligible selection or attrition bias to the reported biomarker outcomes.

### 3.4. Dietary Intake and Nutrient Adequacy

Daily energy and macronutrient intake differed significantly between cases and controls, with significant variations in protein and total calorie consumption. Differences were also observed in other nutrients, including selected vitamins and minerals. Detailed dietary intake data are presented in Table 4.

### 3.5. Distribution of Clinical Symptoms

Cases reported significantly higher frequencies of dermatological, nasal, respiratory, and gastrointestinal symptoms compared with controls. Dermatological manifestations, including itching, redness, dryness, swelling, and bumpy skin texture, were markedly more prevalent among cases. Nasal symptoms such as sneezing, nasal congestion, an itchy nose, and watery eyes were also significantly more frequent among cases.

Respiratory symptoms, including cough, chest tightness, wheezing, and shortness of breath, were reported more frequently by cases. Gastrointestinal symptoms, including diarrhea, constipation, vomiting, heartburn, bloating, and nausea, were significantly more prevalent among cases, whereas reflux and flatulence did not differ significantly between groups. The distribution of reported symptoms is presented in Table 5.

### 3.6. Stress Levels

Assessment using the Perceived Stress Questionnaire indicated that all participants experienced either moderate or high stress levels. In total, 77% of the patients in both groups were classified as having moderate stress, while approximately 22% percent of the participants reported high stress levels. No significant differences in the stress category distribution were observed between cases and controls (Table 6).

### 3.7. Association of Clinical, Biochemical, and Dietary Factors with Food Hypersensitivity

In the multivariable logistic regression analysis, several dietary factors were independently associated with case–control status as shown in Table 7. Higher intakes of carbohydrates (OR = 0.820, 95% CI: 0.717–0.937), fat (OR = 0.682, 95% CI: 0.497–0.936), iron (OR = 0.025, 95% CI: 0.002–0.291), vitamin C (OR = 0.604, 95% CI: 0.390–0.937), vitamin D (OR = 0.079, 95% CI: 0.011–0.565), vitamin E (OR = 0.085, 95% CI: 0.013–0.568), lactose (OR = 0.088, 95% CI: 0.012–0.661), and sugar (OR = 0.516, 95% CI: 0.272–0.979) were independently associated with lower odds of case status.

## 4. Discussion

In the present study, nearly half of the recruited Lebanese adults were classified as having food hypersensitivity, with food allergy being more prevalent than food intolerance and a substantial overlap with IBS (Table 1). This high proportion is consistent with growing global evidence suggesting that food-related disorders are increasingly reported in adult populations, particularly when both immune-mediated allergy and non-immune intolerance are considered together rather than as isolated entities [1,2]. The coexistence of food hypersensitivity with IBS observed in a considerable proportion of cases supports previous findings indicating that food-related immune reactions frequently overlap with functional gastrointestinal disorders, reflecting shared pathophysiological mechanisms such as altered gut permeability, immune activation, and visceral hypersensitivity [16,17,18]. The relatively small number of individuals with isolated IBS in the absence of food hypersensitivity further reinforces the close relationship between these conditions in clinical settings.

A methodological aspect of this study that warrants careful contextualization is the inclusion of microarray-based IgG testing (such as the FOX Food Xplorer) within our laboratory assessment panel. The European Academy of Allergy and Clinical Immunology (EAACI) and other major international allergy consensus guidelines explicitly state that food-specific IgG testing is not clinically validated as a diagnostic tool for food intolerances or allergies, often reflecting normal physiological exposure rather than an immunopathological reaction. However, within the contemporary Lebanese healthcare landscape, food-specific IgG microarrays are widely prescribed by local physicians and clinical dietitians and are frequently sought out directly by symptomatic patients at diagnostic laboratories to manage unexplained chronic gastrointestinal complaints. In this study, IgG microarrays were utilized strictly as an epidemiological mapping tool to characterize the perceived intolerance profiles within this cohort, rather than as a standalone, definitive diagnostic pathology. Researchers and clinicians interpreting these data should treat elevated IgG titers as a proxy marker of frequent dietary exposure and functional gut permeability rather than absolute, prescriptive clinical evidence of food hypersensitivity [1].

Sociodemographic variables, including age, sex distribution, education level, employment status, and marital status, were comparable between cases and controls, suggesting that food hypersensitivity in this population is not primarily driven by socioeconomic or demographic disparities. These findings align with previous studies from Europe and North America, which report that adult food hypersensitivity affects individuals across diverse social and educational backgrounds [6,16]. The geographic distribution across Lebanese governorates further supports the nationwide relevance of food hypersensitivity and suggests that regional dietary habits alone may not fully explain susceptibility, emphasizing the role of individual immune and gastrointestinal factors.

Anthropometric measures, including BMI, did not differ significantly between groups, indicating that food hypersensitivity was not associated with overt differences in body composition, a finding consistent with earlier adult studies reporting normal or comparable BMI in food-allergic and food-intolerant populations [6,19]. In contrast, autoimmune diseases were significantly more prevalent among cases, including rheumatoid arthritis, Hashimoto’s thyroiditis, inflammatory bowel disease (Crohn’s disease and Ulcerative colitis), and psoriasis. This observation supports accumulating evidence of shared immune dysregulation between food hypersensitivity and autoimmune conditions, potentially mediated by impaired oral tolerance, chronic antigen exposure, and sustained gut inflammation [3,17,18]. Smoking was also more prevalent among cases, which may contribute to mucosal barrier dysfunction and immune activation, as tobacco use has been associated with increased intestinal permeability and altered gut microbiota composition [20].

Regarding lifestyle characteristics, a striking finding was that approximately 79–83% of the study population fell into the low physical activity bracket, with no significant differences observed between cases and controls. This high rate of physical inactivity mirrors broader regional epidemiological tracking across urban Lebanon, where walkable infrastructure is highly limited, green spaces are scarce, and negative urbanization forces favor sedentary desk employment or long daily commutes [21]. While physical inactivity is a well-known risk factor for overall metabolic and cardiovascular health, its uniform distribution across both cohorts in this study indicates that it does not act as a specific independent driver or discriminating factor for adult food hypersensitivities in this population.

A striking psychometric finding in this study was the total absence of a ‘low stress’ baseline tier, with 100% of the 775 participants stratifying into either moderate (77.7%) or high (22.3%) stress categories. While such a profound ceiling effect might structurally suggest a limitation in instrument sensitivity or a skewed convenience sample in stable Western contexts, it represents an accurate socio-epidemiological reflection of contemporary Lebanon. During the study recruitment period (July 2024 to June 2025), the Lebanese adult population was enduring an unprecedented, compounding macroeconomic crisis, severe currency devaluation, infrastructure collapse, and severe geopolitical instability. Under these systemic conditions, chronic hyper-vigilance and daily survival stressors act as a collective baseline [22,23,24]. Consequently, the complete shifting of the population variance away from ‘low stress’ highlights the profound environmental validity of the PSQ-30 instrument in capturing a population-wide state of high psychological distress, rather than an analytical artifact or sampling bias.

Despite similar anthropometric profiles, individuals with food hypersensitivity showed significantly lower serum levels of vitamin D (although within the normal range), vitamin B12, hemoglobin, and hematocrit compared with controls. These findings are in line with previous reports indicating that biochemical deficiencies may occur in food-allergic and IBS populations even when dietary intake appears adequate, suggesting roles for malabsorption, chronic low-grade inflammation, or altered intestinal transport mechanisms [3,5,19]. Additionally, the higher prevalence of positive fecal calprotectin among cases indicates intestinal inflammation. Similar patterns have been described in non–IgE-mediated food hypersensitivity and IBS, where low-grade inflammation and microbial imbalance contribute to persistent gastrointestinal and systemic symptoms [21,22,23].

A notable strength of our study is the inclusion of baseline microbiological stool profiling, which provides important proxy insights into the luminal microenvironment despite the logistical and financial absence of high-throughput 16S rRNA gene sequencing. Our findings revealed that individuals with food hypersensitivities had a significantly higher prevalence of positive fungal infections (17.1% vs. 13.9%) and parasitic infections (17.5% vs. 8.1%) compared to healthy controls. In classic models of gastrointestinal homeostasis, a diverse and stable commensal bacterial community provides “colonization resistance,” effectively preventing the overgrowth of opportunistic fungal strains (such as Candida species) and the entrenchment of parasites. The distinct inflation of fungal and parasitic overgrowth among cases strongly signals a state of underlying dysbiosis, where the protective bacterial shield is compromised [25]. Furthermore, this microbiological shift provides a clear mechanistic explanation for the elevated fecal calprotectin levels (23.0%) observed in the case cohort. Fecal calprotectin is a direct biomarker of neutrophil recruitment to the intestinal mucosa. Persistent fungal elements and parasites irritate the intestinal epithelium, triggering an innate immune response that actively recruits neutrophils, induces low-grade mucosal inflammation, and compromises tight junction integrity [26,27]. This ongoing inflammatory cycle alters epithelial transport and digestion, validating our hypothesis that functional gastrointestinal symptoms and altered serum biochemical markers in food hypersensitivity are intimately tied to a disrupted, inflamed gut ecosystem rather than simple dietary elimination alone.

Dietary analysis demonstrated significant differences in daily energy and nutrient intake between cases and controls, with controls consistently reporting higher consumption of total calories, protein, carbohydrates, fat, and most micronutrients. Although both groups generally met or exceeded the recommended dietary allowances (RDAs) for most of the macronutrients and micronutrients, cases exhibited lower intakes compared to controls, particularly for protein, fiber, calcium, iron, potassium, magnesium, zinc, and several vitamins. Notably, fiber intake among cases did not reach the recommended level, which may have implications for gut health and intestinal function. Despite this overall apparent adequacy of dietary intake, individuals with food hypersensitivity presented significant biochemical variations, including lower serum levels of vitamin D, vitamin B12, hemoglobin, and hematocrit. This discrepancy suggests that nutritional impairment in this population may not be solely attributable to insufficient intake, but rather to altered digestion, intestinal inflammation, or impaired nutrient absorption. Similar findings have been reported in both adult and pediatric food-allergic populations, where restrictive dietary patterns and chronic mucosal inflammation contribute to functional malnutrition despite adequate or near-adequate reported intake [3,5,19]. An intriguing finding in our study was the significant association between lower vitamin D status/intake and increased risk of food hypersensitivities, which warrants careful contextualization within the geographic and sociocultural landscape of Lebanon. It is essential to emphasize that dietary supplement consumption was meticulously recorded at baseline and fully integrated into our semi-quantitative Food Frequency Questionnaire (FFQ) nutritional models; thus, the observed deficit cannot be attributed to unmeasured oral supplementation habits. Rather, this highlights a deeply entrenched regional public health phenomenon often referred to as the “Mediterranean Vitamin D Paradox” [28,29]. Despite Lebanon enjoying abundant seasonal sunshine for the majority of the year, hypovitaminosis D remains exceptionally prevalent across the adult population. This high rate of deficiency is largely driven by rapid urbanization and contemporary lifestyle shifts. In dense metropolitan areas such as Greater Beirut, high-rise architectural configurations severely limit ambient daylight penetration, while prevailing employment structures promote prolonged indoor, sedentary desk activity. These structural factors are compounded by high levels of atmospheric pollution in urban centers, which can actively attenuate solar ultraviolet B (UVB) photons required for cutaneous cholecalciferol synthesis [28,29]. Furthermore, cultural, religious, and personal clothing habits that favor extensive body coverage substantially reduce the total surface area of exposed dermal tissue, minimizing natural UV-mediated synthesis even during peak summer months. Consequently, while oral supplement data were rigorously controlled for in our predictive models, natural cutaneous synthesis remains highly constrained by these environmental and lifestyle confounders. Given that vitamin D plays a critical role in preserving intestinal epithelial barrier integrity and modulating mucosal immune tolerance, these regional lifestyle-driven deficits may represent an important environmental cofactor that exacerbates underlying gut–immune hypersensitivity in the Lebanese population [28,29].

A particularly notable finding is the divergence between dietary folate intake, which was significantly lower in the case cohort (*p* = 0.003), and serum folic acid levels, which demonstrated no statistically significant difference between-group variance. This apparent discrepancy can be clinically reconciled by the distinct physiological timelines of these markers. Serum folic acid is highly sensitive to acute homeostatic shifts and fluctuates rapidly in response to recent dietary intake or unrecorded consumption of fortified foods, whereas structural red blood cell parameters and steady-state hemoglobin values (which remained preserved at a healthy mean of approximately 14 g/dL in male participants) reflect long-term hematopoiesis over a 120-day erythrocyte lifespan. Furthermore, the lack of overt macrocytic anemia in the presence of lower dietary folate can be partially attributed to the mandatory national flour fortification programs enacted across the region, which provide baseline tissue protection against severe functional folate deficiencies [30]. This explains how systemic hemoglobin levels remain stable despite a statistically detectable reduction in raw dietary folate intake among individuals managing food hypersensitivities.

Our analysis revealed an inverse association between total sugar intake and food hypersensitivity status. While a cross-sectional timeline requires careful interpretation, this trend is best understood through the lens of pre-diagnostic patient behavior rather than post-diagnostic clinical intervention. Because the dietary metrics captured by the baseline FFQ reflect habits prior to formal clinical evaluation, this lower intake of simple sugars likely represents an empirical, subconscious avoidance strategy by symptomatic individuals. Patients suffering from chronic, undiagnosed gastrointestinal discomfort often self-identify highly processed, hyperosmolar, or refined sugar-rich foods as triggers for functional symptoms like bloating or altered motility. By instinctively minimizing these dietary items to self-manage their systemic discomfort before seeking formal medical consultation, cases present a lower apparent sugar profile compared to healthy controls [31]. This emphasizes that dietary variations in cross-sectional cohorts frequently capture active, pre-diagnostic symptomatic coping mechanisms rather than prescriptive medical elimination patterns.

Collectively, these results highlight the limitations of dietary assessment alone and underscore the importance of incorporating biochemical markers when evaluating nutritional status in individuals with food hypersensitivity.

Cases reported significantly higher frequencies of dermatological, nasal, respiratory, and gastrointestinal symptoms compared with controls, confirming the multisystemic nature of food hypersensitivity. Cutaneous manifestations, including itching, swelling, and dryness, are well-documented features of both IgE- and non–IgE-mediated reactions, which are driven by mast cell activation and inflammatory mediators [18,24]. The high prevalence of nasal and respiratory symptoms supports the concept of a shared mucosal immune system linking the gut, skin, and airways, as previously described in allergic disease models [16,22]. Gastrointestinal symptoms, ranging from diarrhea and vomiting to constipation and bloating, further reflect the heterogeneity of food hypersensitivity presentations and are consistent with reports emphasizing immune–neural and inflammatory mechanisms rather than direct food toxicity alone [18].

Moderate to high perceived stress levels were observed across both groups, highlighting the substantial psychosocial burden within the study population. Chronic stress has been shown to exacerbate gut–brain axis dysfunction, increase intestinal permeability, and modulate immune responses, thereby amplifying symptom severity in food hypersensitivity and IBS [22,24]. Although stress levels did not differ significantly between cases and controls, their overall high prevalence underscores the importance of addressing psychological factors in the comprehensive management of food-related disorders.

The multivariable logistic regression analysis demonstrated that higher intakes of macronutrients (carbohydrates, fats, sugar, and lactose) and selected micronutrients (iron and vitamins C, D, and E) were independently associated with significantly lower odds of being classified as control. These findings suggest that both dietary behavior and nutritional quality play important roles in food hypersensitivity.

The inverse association observed between carbohydrate, fat, sugar, and lactose intake and food hypersensitivity was consistent with previous studies reporting that individuals with food hypersensitivity or food-related gastrointestinal disorders consume significantly lower amounts of energy and macronutrients than healthy individuals. Several studies have shown that patients experiencing chronic gastrointestinal symptoms commonly adopt restrictive eating behaviors and eliminate foods perceived as symptom triggers, often without professional dietary guidance, resulting in lower overall nutrient intake and reduced dietary diversity [30,31,32]. Similarly, international studies have demonstrated that long-term elimination of common allergenic foods, including dairy products and wheat-based foods, was associated with substantially lower intakes of carbohydrates, fats, and total energy unless appropriate nutritional substitutions are implemented [33,34,35]. Therefore, the inverse associations identified in our study likely reflect the consequences of self-imposed dietary restriction rather than a protective effect of these macronutrients themselves.

Higher dietary intakes of vitamins C, D, E, and iron were independently associated with lower odds of food hypersensitivity in the present study. These findings are consistent with previous studies reporting that adequate intake of antioxidant and immunomodulatory micronutrients was associated with a lower prevalence of allergic diseases and improved immune function [3,25,28,29,36,37,38,39,40,41,42]. Previous epidemiological studies have further linked inadequate vitamin D status with an increased risk of food allergy [25,28,29,36], while higher dietary intakes of vitamins C and E have been associated with reduced oxidative stress and inflammatory responses in allergic conditions [3,38,39,40]. Although fewer studies have specifically evaluated dietary iron intake in adults with food hypersensitivity, existing evidence indicates that iron deficiency and lower iron intake are common among individuals following long-term elimination diets or experiencing chronic gastrointestinal disorders [41,42,43].

## 5. Limitations

Several limitations should be considered when interpreting the present findings. First, dietary intake was assessed using a food frequency questionnaire, which is subject to recall bias, reporting errors, and inaccuracies in portion-size estimation and in the type or dosage of supplementation. Secondly, while our continuous convenience sampling strategy drew from an outpatient clinic that serves as a multi-specialty health hub attracting individuals from all Lebanese governorates, the reliance on a single-center clinical cohort introduces a distinct health-seeking selection bias. Because participants aged (18–63), were recruited while proactively presenting for routine health maintenance or annual check-ups, this population may exhibit higher health literacy, distinct dietary behaviors, or increased somatic vigilance compared to the general public. Consequently, this health-seeking disposition may have contributed to an overestimation or inflation of our observed 48.8% food hypersensitivity rate. Although our multi-governorate draws cushions localized geographical bias, these findings should be generalized to the broader Lebanese adult population with caution, highlighting the need for future multi-center, community-based epidemiological surveillance. Another limitation of this study is that information regarding previous self-imposed elimination diets prior to diagnosis was not systematically collected. Some participants may have modified their dietary habits before study enrollment in response to perceived food-related symptoms. Such dietary changes could have influenced nutritional intake and, consequently, certain nutritional and biochemical parameters assessed in this study. Therefore, the potential impact of prior dietary restrictions on the observed findings cannot be excluded. An additional limitation of this study is that Helicobacter pylori testing was not included in the clinical protocol. This omission introduces a potential confounding bias, as chronic H. pylori infection is frequently associated with systemic effects, including weight loss and the malabsorption of critical vitamins and minerals. Consequently, undetected infections among some participants could contribute to the lowered micronutrient profiles observed independently of dietary avoidance behaviors. Another limitation is the reliance on self-reported dietary intake, medication use, and dietary supplement consumption. These data are subject to recall bias, inaccurate reporting, and misclassification, as participants may have had difficulty accurately remembering their habitual food intake, medication use, or supplement consumption. Furthermore, the food frequency questionnaire (FFQ) estimates usual intake over an extended period and therefore may not fully capture temporal variations in dietary habits. Consequently, some degree of measurement error is inevitable, which may have attenuated the observed associations.

## 6. Conclusions

This case–control study provides insight into the systemic nature of food hypersensitivity among Lebanese adults.

By demonstrating strong, independent statistical associations between reduced consumption of essential micronutrients, including antioxidant vitamins (vitamins C and E), vitamin D, and the essential mineral iron, as well as lower intakes of macronutrients and sugars, these findings map out the extensive dietary toll experienced by hypersensitive individuals. Future longitudinal and interventional studies are warranted to clarify causal relationships, further explore the mechanisms linking food hypersensitivity with immune and gastrointestinal dysfunction, and evaluate the effectiveness of integrated nutritional and medical interventions. Such efforts are essential to optimize clinical management and to inform evidence-based guidelines tailored to Middle Eastern populations.

## Figures and Tables

**Table 1:** Classification of food hypersensitivity among cases N (378).

Classification	Participants Frequency (n %)
Cases	378 (48.8)
Allergy	178 (47.1)
Intolerance	82 (21.7)
Allergy and intolerance	13 (3.4)
Allergy and IBS	63 (16.7)
Intolerance and IBS	35 (9.6)
IBS	4(1.1)
Allergy, intolerance, and IBS	3 (0.8)

Note: Data are presented as absolute frequencies (n) and relative percentages (%). Percentages within specific hypersensitivity categories are calculated using the total number of cases (N = 378) as the denominator, while the total case percentage (48.8%) is relative to the entire study cohort (N = 775). Abbreviations: IBS, Irritable Bowel Syndrome.

**Table 2:** Descriptive statistics of the patient’s sociodemographic data (*n* = 775).

Characteristics	Case (*n* = 378) N (%)	Control (*n* = 397) N (%)	*p*-Value	Total (*n* = 775) N (%)
**Gender**				
Male	185 (48.4)	197 (51.6)	0.850	382 (49.3)
Female	193 (49.1)	200 (50.9)		393 (50.7)
**Age (years), mean ± SD**	38.8 ± 0.16	38.9 ± 0.15	0.902	775 (100)
Crowding index *, mean ± SD	1.48 ± 0.17	1.53 ± 0.17	0.259	775 (100)
**Governorate of residence**				
Akkar	15 (4)	24 (6)	0.03	39 (5)
Baalbek–Hermel	21(5.6)	34 (8.7)		55 (7.1)
Beirut	139(36.8)	101 (25.4)		240 (31)
Bekaa	16(4.2)	24 (6)		40 (5.1)
Mount Lebanon	128 (34)	135 (34)		263 (34)
Nabatieh	15 (4)	21 (5.3)		36 (4.6)
North Lebanon	21 (5.6)	26 (6.6)		47 (6.1)
South Lebanon	23 (5.8)	32 (8)		55 (7.1)
**Level of education**				
Elementary School	27 (7.1)	35 (8.8)	0.160	62 (8)
Middle School	46 (12.2)	61 (15.4)		107 (13.8)
High School	46 (12.2)	60 (15.1)		106 (13.7)
University/Higher Education	259 (68.5)	241 (60.7)		500 (64.5)
**Employment status**				
Student	24 (6.3)	28 (7.1)	0.181	52 (6.7)
Not working	155 (41)	130 (32.7)		285 (36.8)
Employed	80(23.8)	100 (25.2)		180 (23.2)
Self-Employed	80 (21.2)	104 (26.2)		184 (23.7)
Retired	29 (7.7)	35 (8.8)		64 (8.2)
**Marital status**				
Single	227 (60.1)	208 (52.4)	0.133	435 (56.1)
Married	144 (38.1)	177 (44.6)		321 (41.4)
Divorced	6 (1.6)	8 (2)		14 (1.8)
Separated	1 (0.3)	1 (0.3)		2 (0.3)
Widowed	0 (0)	3 (0.8)		3 (0.4)

Categorical variables were presented as frequencies and percentages n (%), and continuous variables as mean ± SD. Log10 transformation was used for quantitative variables when they did not follow a normal distribution. Comparisons between case and control groups were performed using the Chi-square test (categorical variables) and the Independent Samples *t*-test (age). A 95% confidence level was applied, and statistical significance was defined as *p* < 0.05. * Crowding index: defined as the number of household members divided by the number of rooms in the household (excluding kitchens and bathrooms); A value ≥ 1 was considered indicative of overcrowding.

**Table 3:** Clinical, lifestyle, and biochemical characteristics of cases and controls.

Characteristics	Case (*n* = 378) N (%)	Control (*n* = 397) N (%)	*p*-Value	Total (*n* = 775) N (%)
**Diseases**				
Hypertension	51 (13.5)	66 (16.6)	0.223	117 (15.1)
Type 2 diabetes	23 (6.1)	25 (6.3)	0.902	48 (6.2)
Dyslipidemia	33 (8.7)	38 (9.6)	0.685	71 (9.2)
Cardiovascular diseases	17 (4.5)	18 (4.5)	0.980	35 (4.5)
Renal Diseases	23 (6.1)	26 (6.5)	0.791	49 (6.3)
Liver Diseases	9 (2.4)	12 (3)	0.582	21 (2.7)
Cancer	7 (1.9)	9 (2.3)	0.685	16 (2.1)
Immunodeficiency	12 (3.2)	16 (4)	0.523	28 (3.6)
Pulmonary diseases	9 (2.4)	12 (3)	0.582	21 (2.7)
Total autoimmune participants (any individual)	66 (17.5)	6 (1.5)	0.000	72 (9.2)
Rheumatoid arthritis	17 (4.5)	2 (0.5)	0.000	19 (2.5)
Hashimoto	15 (4)	2 (0.5)	0.001	17 (2.2)
Lupus	1 (0.3)	0 (0)	0.488	1 (0.12)
Crohn’s disease	15 (4)	0 (0)	0.000	15 (1.93)
Ulcerative colitis	1 (0.3)	0 (0)	0.488	1 (0.12)
Multiple sclerosis	1 (0.3)	0 (0)	0.488	1 (0.12)
Type 1 diabetes	1 (0.3)	0 (0)	0.972	1 (0.12)
Psoriasis	10 (2.6)	1 (0.3)	0.005	11 (1.41)
Grave’s disease	1 (0.3)	0 (0)	0.488	1 (0.1)
Celiac disease	4 (1.1)	0 (0)	0.056	4 (0.5)
Smoking status (yes) *	140 (37)	120 (30.2)	0.000	260 (33.5)
**Type of smoking**				
Argileh (yes)	42 (11.1)	36 (9.1)	0.816	78 (10.06)
Cigarette (yes)	61 (16.1)	50 (12.6)		111 (14.3)
E-Cigarette (yes)	71 (18.8)	48 (19.6)		119 (15.35)
**Physical activity (yes)**				
Vigorous	28 (7.4)	38 (9.6)	0.993	66 (8.5)
Moderate	35 (9.3)	55 (13.8)	0.845	90 (11.6)
**BMI (Kg/m^2^) mean ± SD**	29.13 ± 0.1	28.57 ± 0.1	0.426	775 (100)
**Biochemical testing**				
Hematocrit (%)				
Men	45.1 ± 0.04	45.2 ± 0.04	0.851	
Women	40.7 ± 0.05	41.71 ± 0.03	0.006	
Hemoglobin (g/dL)				
Men	14.54 ± 0.04	14.98 ± 0.03	0.001	
Women	13.51 ± 0.05	13.73 ± 0.03	0.082	
Folic Acid (ng/mL)	9.18± 0.21	9.70 ± 0.20	0.106	
VitB12 (pg/mL)	478.3 ±0.24	516.7 ± 0.21	0.04	
VitD (ng/mL)	52.2 ± 0.29	60.30 ± 0.24	0.001	
Ferritin (ng/mL)				
Men	55.81 ± 0.35	56.08 ± 0.36	0.954	
Women	55.79 ± 0.35	59.56 ± 0.33	0.415	
**Coproculture (stool culture) †**	16 (4.23)	42 (17.2)	0.001	58 (7.48)
Positive bacterial infection				
Positive fungal infection	65 (17.1)	55 (13.9)	0.001	120 (15.48)
Positive parasitic infection	66 (17.5)	32 (8.1)	0.016	98 (12.64)
Positive Calprotectin	87 (23)	32 (8.1)	0.000	119 (15.35)

Categorical variables were presented as frequencies and percentages n (%). Continuous variables are mean ± SD. Log10 transformation was used for quantitative variables when they did not follow a normal distribution. Comparisons between case and control groups were performed using the Chi-square test (categorical variables) and the Independent Samples *t*-test. The Fisher test was used for cells containing fewer than 5 participants. A 95% confidence level was applied, and statistical significance was defined as *p* < 0.05. Reference values: Hemoglobin (men 13–17 g/dL; women 12–16 g/dL); Hematocrit (men 40–52%; women 37–47%); ferritin (men 28–397 ng/mL; women 5–148 ng/mL); vitamin B12 (193–982 pg/mL); folate (4–20 ng/mL); vitamin D (27–120) ng/mL. * Percentages for individual tobacco categories sum to >37% due to the presence of poly-tobacco use (e.g., simultaneous cigarette and argileh smoking) within the case cohort. † Within the total study sample (N = 775), a specific sub-cohort of 297 participants (representing 38.3% of the total population) underwent formal stool analysis.

**Table 4:** Comparison of daily energy and nutrient intake between cases, controls, and the RDA.

Characteristic	Cases (*n* = 378) Mean ± SD	Controls (*n* = 397) Mean ± SD	RDA	*p*-Value (Case vs. Control)	*p*-Value (Case vs. RDA)	*p*-Value (Control vs. RDA)	Total (*n* = 775) N (%)
Energy (kcal/day)	2339 ± 0.2	2535 ± 0.2	2500 (M), 2000 (F)	0.003	<0.001	<0.001	775 (100)
Male	2359 ± 0.2			<0.001			
Female	2320 ± 0.2			0.810			
Protein (g/day)	53 ± 0.2	58 ± 0.2	50	<0.001	<0.001	<0.001	
Carbohydrates (g/day)	285 ± 0.2	314 ± 0.2	275	<0.001	<0.001	<0.001	
Fat (g/day)	81 ± 0.1	89 ± 0.2	70–78	<0.001	<0.001	<0.001	
Calcium (mg/day)	1043 ± 0.2	1139 ± 0.04	1000	0.001	<0.001	<0.001	
Iron (mg/day)	12.6 ± 0.2	13.7 ± 0.2	12	0.023	<0.001	<0.001	
Potassium (mg/day)	3094 ± 0.2	3358 ± 0.2	3000	0.002	<0.001	<0.001	
Sodium (mg/day)	1549 ± 0.02	1552 ± 0.02	1500	0.644	<0.001	<0.001	
Vitamin A (µg/day)	797 ± 0.04	799 ± 0.04	800	0.714	0.984	0.467	
Vitamin C (mg/day)	86 ± 0.2	94 ± 0.2	82	<0.001	<0.001	<0.001	
Vitamin D (µg/day)	16 ± 0.1	17 ± 0.2	15	0.001	<0.001	<0.001	
Vitamin E (mg/day)	16 ± 0.2	17 ± 0.2	15	0.001	<0.001	<0.001	
Vitamin K (µg/day)	109 ± 0.2	118 ± 0.2	105	0.002	<0.001	<0.001	
Folate (µg/day)	419 ± 0.2	452 ± 0.2	400	0.003	<0.001	<0.001	
Vitamin B12 (µg/day)	2.5 ± 0.2	2.8 ± 0.2	2.4	<0.001	<0.001	<0.001	
Magnesium (mg/day)	367 ± 0.2	400 ± 0.2	350	0.001	<0.001	<0.001	
Zinc (mg/day)	9.7 ± 0.2	10.7 ± 0.2	9.5	0.001	<0.001	<0.001	
Lactose (g/day)	12.4 ± 0.2	13.6 ± 0.2	12	<0.001	<0.001	<0.001	
Sugar (g/day)	32 ± 0.2	34 ± 0.2	25–36	0.007	<0.001	<0.001	
Fiber (g/day)	29 ± 0.2	31 ± 0.2	30	0.005	0.212	<0.001	

Continuous variables were presented as mean ± SD. Log10 of continuous variables was used when they did not follow a normal distribution. All inferential statistics (*p*-values) were performed on log-transformed data. For interpretability, back-transformation of mean values corresponds to geometric means (10^mean log values), while variability corresponds to multiplicative dispersion rather than arithmetic standard deviations, and comparisons between cases and controls were performed using independent samples tests. Comparison of daily nutrients intake with the Recommended Dietary Allowances (RDA) [15] was performed using a one-sample *t*-test. Categorical variables were presented as n (%), with comparisons performed using Chi-square tests. *p* < 0.05 was considered statistically significant.

**Table 5:** Prevalence of skin, nasal, respiratory, life-threatening, and gastrointestinal symptoms among case and control groups.

Characteristics	Case (*n* = 378) N (%)	Control (*n* = 397) N (%)	*p*-Value	Total (*n* = 775) N (%)
**Skin symptoms**				
Itching	130 (34.4)	59 (14.9)	0.000	189 (24.4)
Redness	145 (38.4)	45 (11.3)	0.000	190 (24.5)
Bumpy texture	170 (45)	59 (14.9)	0.000	229 (29.5)
Dryness	169 (44.7)	59 (14.9)	0.000	228 (29.4)
Swelling	161 (42.6)	61 (15.4)	0.000	222 (28.6)
**Nasal symptoms**				
Nasal congestion, or rub	181 (39.7)	31 (7.8)	0.000	181 (23.4)
Bad breath	165 (43.7)	73 (18.4)	0.000	238 (30.7)
Headaches	254 (67.2)	243 (61.2)	0.085	497 (64.1)
Fatigue/irritability	168 (44.4)	156 (39.3)	0.166	324 (41.8)
Red eyes	169 (44.7)	15 (3.8)	0.000	184 (23.7)
Snoring	151 (39.9)	15 (3.8)	0.000	166 (21.4)
Itchy eyes	170 (45)	31 (7.8)	0.000	201 (25.9)
Mouth breathing, or sneezing	179 (47.4)	46 (11.6)	0.000	225 (29)
Runny nose	171 (45.2)	32 (8.1)	0.000	203 (26.2)
Sinus infection	174 (46)	76 (19.1)	0.000	250 (32.3)
Mucopurulent nasal discharge	169 (44.7)	122 (30.7)	0.000	291 (37.5)
Loss of taste and smell	164 (43.4)	106 (26.7)	0.000	270 (34.8)
**Respiratory symptoms**				
Cough	174 (46)	29 (7.3)	0.000	203 (26.2)
Cough from postnasal drip	142 (37.6)	16 (4)	0.000	158 (20.4)
Tightness	92 (35.7)	16 (4)	0.000	151 (19.5)
Quincke edema	38 (10.1)	15 (3.8)	0.001	53 (6.8)
**Gastric symptoms**				
**Upper gastrointestinal symptoms**				
Vomiting	52 (13.7)	16 (4)	0.000	68 (8.8)
Heartburn	85 (22.5)	45 (11.3)	0.000	130 (16.8)
Reflux	107 (28.3)	122 (30.7)	0.479	229 (29.5)
Nausea	96 (25.4)	32 (8.1)	0.000	128 (16.5)
**Lower gastrointestinal symptoms**				
Diarrhea	76 (20.1)	31 (7.8)	0.000	107 (13.8)
Constipation	91 (24.1)	62 (15.6)	0.004	153 (19.7)
Bloating	157 (41.5)	123 (31)	0.003	280 (36.1)
Flatulence	116 (30.6)	124 (31.2)	0.877	240 (31)

Categorical variables were presented as frequencies and percentages n (%). Comparisons between case and control groups were performed using the Chi-square test (categorical variables) and the Independent Samples *t*-test (age). A 95% confidence level was applied, and statistical significance was defined as *p* < 0.05.

**Table 6:** Categorized stress level PSQ.

Characteristics	Case (*n* = 378) N (%)	Control (*n* = 397) N (%)	*p*-Value	Participants Frequency n (%)
Low and Moderate	293 (77.5)	309 (77.7)	0.831	602 (77.7)
High	85 (22.4)	88 (22.1)		173 (22.3)

Categorical variables were presented as frequencies and percentages n (%). Comparisons between case and control groups were performed using the Chi-square test (categorical variables). A 95% confidence level was applied, and statistical significance was defined as *p* < 0.05.

**Table 7:** Multivariable binary analyses cases vs. control N (775).

Variable	OR	95% CI
Carbohydrate	0.820	0.717–0.937
Fat	0.682	0.497–0.936
Iron	0.025	0.002–0.291
Vitamin C	0.604	0.390–0.937
Vitamin D	0.079	0.011–0.565
Vitamin E	0.085	0.013–0.568
Lactose	0.088	0.012–0.661
Sugar	0.516	0.272–0.979

**Abbreviations:** OR, odds ratio; CI, confidence interval. Outcome coding: Control = 1 and Case = 2; therefore, ORs > 1 indicate increased odds of case status, whereas ORs < 1 indicate decreased odds of case status. Adjusted model: The multivariable logistic regression model was adjusted for age, gender, governorate of residence (all Lebanese governorates compared with South Lebanon as the reference category), smoking status (Yes = 1, No = 2), BMI, hematocrit serum level, vitamin B12 serum level, and dietary intake variables that were statistically significant in the bivariate analysis. Model diagnostics: The model was statistically significant (Omnibus χ^2^ = 1038.233, *df* = 31, *p* < 0.001), demonstrated excellent calibration according to the Hosmer–Lemeshow goodness-of-fit test (χ^2^ = 0.025, *df* = 8, *p* = 1.000), and explained a substantial proportion of the variance in case–control status (Nagelkerke R^2^ = 0.984; Cox and Snell R^2^ = 0.738). The overall classification accuracy was 99.0%.

## Data Availability

The data presented in this study are available on request from the corresponding author due to data protection restrictions and ethical constraints.

## References

[1] Hage G., Sacre Y., Haddad J., Hajj M., Sayegh L.N., Fakhoury-Sayegh N. (2025). Food Hypersensitivity: Distinguishing Allergy from Intolerance, Main Characteristics, and Symptoms—A Narrative Review. Nutrients.

[2] Loh W., Tang M.L.K. (2018). The Epidemiology of Food Allergy in the Global Context. Int. J. Environ. Res. Public Health.

[3] Vassilopoulou E., Venter C., Roth-Walter F. (2024). Malnutrition and Allergies: Tipping the Immune Balance towards Health. J. Clin. Med..

[4] Meyer R., De Koker C., Skrapac A.-K., Godwin H., Reeve K., Chebar-Lozinsky A., Shah N. (2015). A Practical Approach to Vitamin and Mineral Supplementation in Food Allergic Children. Clin. Transl. Allergy.

[5] Sova C., Feuling M.B., Baumler M., Gleason L., Tam J.S., Zafra H., Goday P.S. (2013). Systematic Review of Nutrient Intake and Growth in Children with Multiple IgE-Mediated Food Allergies. Nutr. Clin. Pract..

[6] Skypala I.J., Taylor C.F., Pallister A., Scadding G.W. (2021). A Pilot Study to Evaluate the Dietary Intake of Adults Attending a Food Allergy Clinic, and Compare the Results Against the Final Diagnostic Outcome. Front. Allergy.

[7] Kaganov B., Caroli M., Mazur A., Singhal A., Vania A. (2015). Suboptimal Micronutrient Intake among Children in Europe. Nutrients.

[8] Kotchetkoff E.C.d.A., de Oliveira L.C.L., Sarni R.O.S. (2024). Elimination Diet in Food Allergy: Friend or Foe?. J. Pediatr. (Rio J.).

[9] Jomaa L., Hwalla N., Itani L., Chamieh M.C., Mehio-Sibai A., Naja F. (2016). A Lebanese Dietary Pattern Promotes Better Diet Quality among Older Adults: Findings from a National Cross-Sectional Study. BMC Geriatr..

[10] Chafei H., El Harake M.D., Toufeili I., Kharroubi S.A. (2023). Knowledge, Attitudes, and Practices of Consumers on Food Allergy and Food Allergen Labeling: A Case of Lebanon. Foods.

[11] Craig C.L., Marshall A.L., Sjöström M., Bauman A.E., Booth M.L., Ainsworth B.E., Pratt M., Ekelund U., Yngve A., Sallis J.F. (2003). International Physical Activity Questionnaire: 12-Country Reliability and Validity. Med. Sci. Sports Exerc..

[12] Levenstein S., Prantera C., Varvo V., Scribano M.L., Berto E., Luzi C., Andreoli A. (1993). Development of the Perceived Stress Questionnaire: A New Tool for Psychosomatic Research. J. Psychosom. Res..

[13] Razzaq H.M. (2023). Reliability And Validity of Thearabic Version of The Perceived Stress Questionnaire (PSQ). HIV Nurs..

[14] Drossman D.A., Dumitrascu D.L. (2006). Rome III: New Standard for Functional Gastrointestinal Disorders. J. Gastrointest. Liver Dis..

[15] Aoun C., Bou Daher R., El Osta N., Papazian T., Khabbaz L.R. (2019). Reproducibility and Relative Validity of a Food Frequency Questionnaire to Assess Dietary Intake of Adults Living in a Mediterranean Country. PLoS ONE.

[16] Kasırga E. (2019). The Importance of Stool Tests in Diagnosis and Follow-up of Gastrointestinal Disorders in Children. Turk. Arch. Pediatr..

[17] USDA/HHS U.S. Department of Agriculture & U.S. Department of Health and Human Service (2020). Dietary Guidelines for Americans, 2020–2025.

[18] Tedner S.G., Asarnoj A., Thulin H., Westman M., Konradsen J.R., Nilsson C. (2022). Food Allergy and Hypersensitivity Reactions in Children and Adults—A Review. J. Intern. Med..

[19] Muraro A., Halken S., Arshad S.H., Beyer K., Dubois A.E.J., Du Toit G., Eigenmann P.A., Grimshaw K.E.C., Hoest A., Lack G. (2014). EAACI Food Allergy and Anaphylaxis Guidelines. Primary Prevention of Food Allergy. Allergy.

[20] Connors L., O’Keefe A., Rosenfield L., Kim H. (2018). Non-IgE-Mediated Food Hypersensitivity. Allergy Asthma Clin. Immunol..

[21] D’Auria E., Pendezza E., Leone A., Riccaboni F., Bosetti A., Borsani B., Zuccotti G., Bertoli S. (2022). Nutrient Intake in School-Aged Children with Food Allergies: A Case-Control Study. Int. J. Food Sci. Nutr..

[22] Conlon M.A., Bird A.R. (2014). The Impact of Diet and Lifestyle on Gut Microbiota and Human Health. Nutrients.

[23] Sibai A.M., Costanian C., Tohme R., Assaad S., Hwalla N. (2013). Physical Activity in Adults with and without Diabetes: From the “high-Risk” Approach to the “Population-Based” Approach of Prevention. BMC Public Health.

[24] World Health Organization (2025). Public Health Situation Analysis (PHSA): Lebanon—July 2025.

[25] Caballero-Flores G., Pickard J.M., Núñez G. (2023). Microbiota-Mediated Colonization Resistance: Mechanisms and Regulation. Nat. Rev. Microbiol..

[26] Atoui M., Farran D., Mobarak H., Chamoun Y., El Dirani E., Zeinoun L., El Khoury J., Zarzour M. (2025). Adapting Psychiatric Practice in War Zones: Clinicians Experience from the Lebanon 2024 War. Geopsychiatry.

[27] Vereinte Nationen, UNDP, Internationale Arbeitsorganisation, UNICEF, UN-Habitat (2025). The Socioeconomic Impacts of the 2024 War on Lebanon.

[28] Underhill D.M., Braun J. (2022). Fungal Microbiome in Inflammatory Bowel Disease: A Critical Assessment. J. Clin. Investig..

[29] Meng X., Zhang G., Cao H., Yu D., Fang X., de Vos W.M., Wu H. (2020). Gut Dysbacteriosis and Intestinal Disease: Mechanism and Treatment. J. Appl. Microbiol..

[30] Al-Jawaldeh A., Taktouk M., Doggui R., Abdollahi Z., Achakzai B., Aguenaou H., Al-Halaika M., Almamary S., Barham R., Coulibaly-Zerbo F. (2021). Are Countries of the Eastern Mediterranean Region on Track towards Meeting the World Health Assembly Target for Anemia? A Review of Evidence. Int. J. Environ. Res. Public Health.

[31] Pesce M., Cargiolli M., Cassarano S., Polese B., De Conno B., Aurino L., Mancino N., Sarnelli G. (2020). Diet and Functional Dyspepsia: Clinical Correlates and Therapeutic Perspectives. World J. Gastroenterol..

[32] Vassilopoulou E., Feketea G., Konstantinou G.N., Zekakos Xypolias D., Valianatou M., Petrodimopoulou M., Vourga V., Tasios I., Papadopoulos N.G. (2022). Food Protein-Induced Allergic Proctocolitis: The Effect of Maternal Diet During Pregnancy and Breastfeeding in a Mediterranean Population. Front. Nutr..

[33] Goyal O., Goyal M.K., Gupta A., Batta S., Singh A., Goyal P., Mehta V., Sood A. (2025). Self-Reported Food Triggers and Food Fears Impact Nutrient Intake and Quality of Life in Patients with Irritable Bowel Syndrome and Functional Dyspepsia. Korean J. Gastroenterol..

[34] Venter C., Meyer R. (2010). Session 1: Allergic Disease The Challenges of Managing Food Hypersensitivity: Symposium on ‘Dietary Management of Disease’. Proc. Nutr. Soc..

[35] Mehta H., Groetch M., Wang J. (2013). Growth and Nutritional Concerns in Children with Food Allergy. Curr. Opin. Allergy Clin. Immunol..

[36] Gannagé-Yared M.-H., Chemali R., Yaacoub N., Halaby G. (2000). Hypovitaminosis D in a Sunny Country: Relation to Lifestyle and Bone Markers. J. Bone Miner. Res..

[37] Bassil D., Rahme M., Hoteit M., Fuleihan G.E.-H. (2013). Hypovitaminosis D in the Middle East and North Africa. Dermato-Endocrinology.

[38] Maslin K., Venter C., MacKenzie H., Vlieg-Boerstra B., Dean T., Sommer I. (2018). Comparison of Nutrient Intake in Adolescents and Adults with and without Food Allergies. J. Hum. Nutr. Diet..

[39] Vollbracht C., Raithel M., Krick B., Kraft K., Hagel A.F. (2018). Intravenous Vitamin C in the Treatment of Allergies: An Interim Subgroup Analysis of a Long-Term Observational Study. J. Int. Med. Res..

[40] Amevor F.K., Cui Z., Du X., Ning Z., Deng X., Xu D., Shu G., Wu Y., Cao X., Shuo W. (2022). Supplementation of Dietary Quercetin and Vitamin E Promotes the Intestinal Structure and Immune Barrier Integrity in Aged Breeder Hens. Front. Immunol..

[41] Cook-Mills J.M., Averill S.H., Lajiness J.D. (2022). Asthma, Allergy and Vitamin E: Current and Future Perspectives. Free Radic. Biol. Med..

[42] Bhattacharyya A., Chattopadhyay R., Mitra S., Crowe S.E. (2014). Oxidative Stress: An Essential Factor in the Pathogenesis of Gastrointestinal Mucosal Diseases. Physiol. Rev..

[43] Anwar S., Alrumaihi F., Sarwar T., Babiker A.Y., Khan A.A., Prabhu S.V., Rahmani A.H. (2024). Exploring Therapeutic Potential of Catalase: Strategies in Disease Prevention and Management. Biomolecules.

